# ADAR1 prevents ZBP1-dependent PANoptosis via A-to-I RNA editing in developmental sevoflurane neurotoxicity

**DOI:** 10.1007/s10565-024-09905-1

**Published:** 2024-07-25

**Authors:** Huiling Yang, Sen Xu, Xinya Hong, Yusi Liu, Shaojie Qian, Yifei Lou, Wenyuan Wang

**Affiliations:** 1 Department of Anesthesiology, Affiliated Hangzhou Xixi Hospital, Zhejiang Chinese Medical University, Hangzhou, 310023 Zhejiang China; 2 Zhejiang Chinese Medical University, Hangzhou, 310053 Zhejiang China; 3 Center for Rehabilitation Medicine, Department of Anesthesiology, Zhejiang Provincial People’s Hospital, Affiliated People’s Hospital, Hangzhou Medical College, Hangzhou, 310014 Zhejiang China

**Keywords:** Sevoflurane, PANoptosis, ADAR1, ZBP1, RNA editing, Developing brain

## Abstract

It is well established that sevoflurane exposure leads to widespread neuronal cell death in the developing brain. Adenosine deaminase acting on RNA-1 (ADAR1) dependent adenosine-to-inosine (A-to-I) RNA editing is dynamically regulated throughout brain development. The current investigation is designed to interrogate the contributed role of ADAR1 in developmental sevoflurane neurotoxicity. Herein, we provide evidence to show that developmental sevoflurane priming triggers neuronal pyroptosis, apoptosis and necroptosis (PANoptosis), and elicits the release of inflammatory factors including IL-1β, IL-18, TNF-α and IFN-γ. Additionally, ADAR1-P150, but not ADAR1-P110, depresses cellular PANoptosis and inflammatory response by competing with Z-DNA/RNA binding protein 1 (ZBP1) for binding to Z-RNA in the presence of sevoflurane. Further investigation demonstrates that ADAR1-dependent A-to-I RNA editing mitigates developmental sevoflurane-induced neuronal PANoptosis. To restore RNA editing, we utilize adeno-associated virus (AAV) to deliver engineered circular ADAR-recruiting guide RNAs (cadRNAs) into cells, which is capable of recruiting endogenous adenosine deaminases to promote cellular A-to-I RNA editing. As anticipated, AAV-cadRNAs diminishes sevoflurane-induced cellular Z-RNA production and PANoptosis, which could be abolished by ADAR1-P150 shRNA transfection. Moreover, AAV-cadRNAs delivery ameliorates developmental sevoflurane-induced spatial and emotional cognitive deficits without influence on locomotor activity. Taken together, these results illustrate that ADAR1-P150 exhibits a prominent role in preventing ZBP1-dependent PANoptosis through A-to-I RNA editing in developmental sevoflurane neurotoxicity. Application of engineered cadRNAs to rectify the compromised ADAR1-dependent A-to-I RNA editing provides an inspiring direction for possible clinical preventions and therapeutics.

**Supplementary Information:**

The online version contains supplementary material available at 10.1007/s10565-024-09905-1.

## Introduction

General anaesthesia is usually considered to provide a safe and reversible brain state for the performance of surgery. In the developing brain, however, a growing body of experimental evidence intimates that anaesthetic drugs can trigger widespread neuronal loss and subsequent long-term neurocognitive alterations (Vutskits and Xie 2016). Recent clinical findings also suggest lasting effects of early life exposure to anesthetics on brain structures and later behavioral problems (Salaun et al. 2023; Ing et al. 2021). During this critical period of neurodevelopment, pharmacological interference with these physiological process can exert lasting negative effects on brain function. Given that many pregnant women, newborns and infants are clinically exposed to general anesthetics, it is urgent to investigate the mechanism underlying the developmental anesthetic neurotoxicity.

ADAR1 (adenosine deaminase acting on RNA-1) is a member of ADAR family, and is abundantly expressed in the developing brain. Loss of ADAR1 leads to embryonic lethality (Hartner et al. 2009), emphasizing an essential role in neurodevelopment and cell survival. ADAR1 deficiency and the subsequent inflammatory response have been associated with several autoinflammatory and neurodegenerative disorders, such as Aicardi-Goutières syndrome, bilateral striatal necrosis, and systemic lupus erythematosus (Rice et al. 2012; Livingston et al. 2014; Roth et al. 2018). As to ADAR1 isoforms, the ADAR1-P150 isoform shuttles between the nucleus and cytoplasm due to the presence of nuclear export signal and nuclear localization sequence, while the ADAR1-P110 isoform that contains only nuclear localization sequence predominantly localizes in the nucleus (Karki and Kanneganti 2023). One of the key unique structural features of ADAR1-P150 is its Zα domain, which is reported to sense Z-nucleic acids (Z-NAs). The only other molecule that possesses a Zα domain in mammals is the Z-DNA/RNA binding protein 1 (ZBP1, also called DAI or DLM1), which is initially recognized as an interferon-inducible Z-NA binding protein (Kuriakose and Kanneganti 2018). However, several recent studies suggest that there is a regulatory connection between ZBP1 and ADAR1. For instance, ablation of ZBP1 rescues the overt pathology caused by ADAR1 mutation (Hubbard et al. 2022).

Adenosine-to-Inosine (A-to-I) RNA editing by the ADARs is the most prevalent form of RNA modification in mammals (Riella et al. 2022). Editing of specific adenosines may modify the sequence and function of the protein product, as inosines are interpreted usually as guanosines. A critical role of A-to-I RNA editing is the ability to prevent false activation of the innate immune system triggered by endogenous double-stranded RNAs (dsRNAs). The A-to-I RNA editing levels are dynamically regulated throughout brain development which peaks in the period of mid-fetal and infancy development (Hwang et al. 2016; Cuddleston et al. 2022a, b). Recently, ZBP1 is considered as a dsRNA sensor, which can recognize dsRNAs and then elicit a detrimental inflammatory response (Wolf and Lee-Kirsch 2022). Consistent with these important biological functions, alterations in ADARs expression and RNA editing are linked to several neurodevelopmental and neuropsychiatric disorders, including autism, epilepsy and schizophrenia (Breen et al. 2019; Tran et al. 2019; Brusa et al. 1995). However, the contributed role of ADARs-dependent A-to-I RNA editing in developmental anesthetic neurotoxicity remains to be identified.

In the previous study, we have shown that developmental sevoflurane exposure gives rise to aberrant ZBP1 activation and inflammatory response (Wang et al. 2022). In the present study, we tested the hypothesis that ADAR1-P150 dependent A-to-I RNA editing restricts aberrant ZBP1 activation and cellular PANoptosis (Pyroptosis, Apoptosis and Necroptosis) in developmental sevoflurane neurotoxicity. Characterizing these processes will bring new insights into understanding the mechanism of developmental anesthetic neurotoxicity and provide evidence for possible clinical preventions and therapeutics.

## Materials and methods

### Animals experiments and reagents

All animal experiments were approved by the Institutional Animal Care and Use Committee (Zhejiang Provincial People’s Hospital, Affiliated People`s Hospital Hangzhou Medical College, Hangzhou, China), and undertaken in accordance with the National Institute of Health Guide for the Care and Use of Laboratory Animals. The Sprague–Dawley rats were acquired from China Academy of Chinese Medical Sciences (Shanghai, China). All rats were housed under specific-pathogen-free conditions with food and water available ad libitum. All rats were maintained at 22–24 °C and ~ 55% humidity, with a normal 12-h light/dark cycle during the experiments. Adeno-associated virus (AAV) packaged circular ADAR-recruiting guide RNAs (cadRNAs) plasmid were obtained from W. Wei’s laboratory (School of Life Sciences, Peking University). AAV-cadRNAs were injected retro-orbitally into each rat pup, at a dose of 1.0 × 10^13^ vector genomes per rat. To knock down ZBP1 expression in vivo, RNA interference was applied using the short hairpin RNA (shRNA) against ZBP1. To enhance ADAR1 P150 expression in vivo, ADAR1-P150 overexpression plasmids (pmGFP-ADAR1-P150, Addgene plasmid) was transfected. Briefly, the in vivo transfection of plasmid or shRNA was performed according to the manufacturer's instructions (Engreen, Shanghai, China). The pmGFP-ADAR1-P150 plasmid or ZBP1 shRNA was added to Entranster‐in vivo transfection reagents, and then mixed for 15 min at 25℃. Entranster-in vivo mixture was injected intracerebroventricularly 48 h prior to sevoflurane treatment. Efforts were made to minimize the number and suffering of animals. The reagents were obtained from Abcam (Hong Kong, China), Gibco Invitrogen (Carlsbad, USA), Sigma-Aldrich (St. Louis, USA), Cell Signaling Technology (Beverly, USA), Santa Cruz Biotechnology (Santa Cruz, USA) and Beyotime (Shanghai, China).

### Neuronal cultures and cell transfection

Hippocampal neurons were obtained from Sprague Dawley rat embryonic day 18 as previously described. In brief, the hippocampal tissues were collected from rat embryos and digested with 0.25% trypsin. The cells were plated onto coverslips, dishes or 96-well plates at the concentration of 5 × 10^4^ per milliliter. Neuronal cultures were fed with Neurobasal media combined with 2% of B27 and 0.5 mM of L-glutamine, and maintained in a humidified atmosphere of 95% air and 5% CO_2_ at 37 °C. Half of the medium was replaced with fresh medium every 2–3 days. To knock down specific signals, RNA interference was applied using the shRNA against ADAR1-P150 or ZBP1. To enhance ADAR1 isoforms expression, ADAR1-P150 or ADAR1-P110 overexpression plasmids (pmGFP-ADAR1-P150 or pmGFP-ADAR1-P110, Addgene plasmid) was transfected for 48 h. At 5 days in vitro (DIV), hippocampal neurons were transfected with Lipofectamine 3000 on six-well plates according to the manufacturer’s instructions. Before sevoflurane priming at 7 DIV, half of the medium was replaced with fresh Neurobasal medium.

### Sevoflurane exposure

Rat pups and hippocampal neurons were exposed to sevoflurane as previously described (Wen-Yuan et al. 2023). Briefly, the rat pups were treated with sevoflurane delivered by 50% O_2_ combined with 50% N_2_. The gas mixture was provided from a calibrated vaporizer at a flow rate of 2 L/min. The hippocampal neurons were exposed to sevoflurane delivered by a humidified gas mixture of 5% CO_2_ and 95% air at a flow rate of 1 L/min. Control experiments were conducted in the same manner, except no sevoflurane was delivered. The concentrations of O_2_, CO_2_, N_2_ and sevoflurane were monitored continuously by a gas analyzer (Datex Ohmeda, USA). After sevoflurane priming, the rat pups were killed by decapitation for the subsequent experiments. Other rat pups were housed for behavior study 4 weeks later.

### Subcellular fractionation

The subcellular fractionation was performed as previously described (Evavold et al. 2021). Briefly, the neuronal cultures were collected after sevoflurane treatment. The cells were centrifuged at 400 g for 3 min at 4 ℃. The pellet was then resuspended in hypotonic buffer followed by incubation on ice for 30 min. Homogenizing treatment was performed by passing cells for 50 times through a 26-gauge needle. Cell debris and nuclei were precipitated by centrifugation at 2,500 g, for 5 min at 4 ℃. The supernatants were then centrifuged at 17,000 g for 30 min at 4 ℃ to precipitate cellular membranes. The supernatant was cytosolic fraction. The membrane pellet was washed with hypotonic buffer for three times. For whole-cell protein extraction, the primary hippocampal neuronal cultures were subjected to lysis buffer. Whole-cell lysates were centrifuged at 12,000 g for 5 min 4 °C, and the supernatant was collected for immunoblot analysis.

### Immunoblot and immunoprecipitation

Cells and tissues were lysed in RIPA buffer according to the manufacturer’s instruction. The protein concentrations were measured by Bradford Protein Assay (Bio-Rad, Hercules, California, USA). 20 ug of cell lysates were separated by SDS-PAGE and transferred to a PVDF membrane. The PVDF membrane was incubated with blocking buffer at 25℃ for 30 min, and then incubated with appropriate primary antibody (anti-ADAR1-P150 (1:1,000), anti-ADAR-P110 (1:1000), anti-ADAR2 (1:1,000), anti-RIPK3 (1:1000), anti-P-MLKL (1:1000), anti-ZBP1 (AdipoGen, 1:1000), anti-GSDMD-NT (1:1000), anti-GSDME-NT (1:1000), anti-cleaved Caspase-7 (1:1000), anti-cleaved Caspase-3 (1:1000), anti-NINJ1 (1:1000), anti-HMGB1 (1:1000), anti-Z-NAs (Z22 clone, Absolute antibody, 1:1000)) followed by secondary antibody (IgG-HRP–conjugated secondary antibody (1:2000)). The anti-Actin (1:2000) and anti-voltage-dependent anion channel 1 (VDAC1, 1:1000) served as control markers for the cytosol fraction and membrane, respectively. ECL kit (Bio-Rad) was used to develop signals. As to immunoprecipitation, the cell lysates were precipitated with 1 μg of anti-ADAR1, anti-ZBP1 or IgG control antibody, and incubated overnight at 4 °C with protein A/G PLUS-Agarose. The beads were washed with 1 mL of M2 buffer for five times. The bound proteins were boiled in SDS buffer and separated by SDS-PAGE.

### Immunofluorescence imaging

Following treatment, neuronal cultures were fixed with 4% (w/v) paraformaldeyde and permeabilized by 0.5% (v/v) Triton X-100 in PBS at room temperature. Neurons were blocked by 10% goat serum at 25℃, and then incubated overnight with anti-MAP2 (1:1000) or anti-Z-NAs (Z22 clone, 1:200) at 4 °C. After three washes in PBS, the goat anti-rabbit coupled to FITC (1:200) or goat anti-mouse coupled to TRITC (1:200) was incubated for 2 h at 25℃. Nuclei were stained with DAPI. The slides were imaged by confocal microscope (Zeiss, Germany) following three washes in PBS.

### Assessment of cell viability

Cell viability was assessed by ToxiLight Non-destructive Cytotoxicity BioAssay Kit (Lonza, Basel, Switzerland) according to the manufacture's instructions. The activity of adenylate kinase was determined as the number of lytic cells. Briefly, cells were seeded at a density of 2.5 × 10^4^ cells per well in a 96-well plate. After priming with sevoflurane, 50 μLof the ToxiLight assay buffer was added and incubated under shaking for 5 min. The luminescence was then measured using a multi-mode plate reader (Bio Tek, USA). PI-staining was performed according to the manufacture’s instructions. The release of lactate dehydrogenase (LDH) was also used to evaluate cell death. After treatment with sevoflurane, the reconstituted substrate mix (60 μL) was added, and then incubated at 25℃ for 30 min in dark. The absorbance was determined at 490 nm.

### Flow cytometry and ELISA assay

After sevoflurane priming (3%, 6 h), hippocampal neurons were stained with Fixable Viability Stain 700 (BD, USA) and permeabilized with Cytofix/Cytoperm (BD, USA) according to the manufacture's instructions. Following DNase I treatment, cells were blocked and incubated with anti-Z-NA antibody Z22 clone (1:100) at 4 °C for 30 min. The cells were then washed three times with Perm/WashTM Buffer (BD, USA) followed by incubation with anti-mouse-IgG 488 antibody (Invitrogen, USA). The samples were evaluated by a BD LSRFortessa Cell Analyzer to determine the percentage of Z-NA positive cells. The levels of TNF-α, IL-18, IL-1β and IFN-γ were examined by using an ELISA kit according to the instructions of manufacturer. After sevoflurane priming (3%, 6 h), the medium of neuronal cultures was collected and then added to 96-well plates which were precoated with the indicated antibodies. The absorbance values were examined at 450 nm.

### RIP and Quantitative RT-PCR

RNA immunoprecipitation (RIP) was carried out using a Magna RIP RNA-Binding Protein Immunoprecipitation kit (Millipore, USA) according to the instructions of manufacturer. In brief, hippocampal neurons were harvested and lysed in RIP Lysis Buffer with RNase and protease inhibitors. The lysate was subjected to immunoprecipitation using anti-ADAR1, anti-ZBP1 or anti-Z-NAs (Z22 clone) antibody. Samples were then incubated with proteinase K, and the immunoprecipitated RNA was extracted using phenol:chloroform:isoamyl alcohol, followed by quantitative RT-PCR. Total RNA was extracted using Trizol reagent, and cDNA was synthesized by PrimeScript RT reagent Kit (TaKaRa, Japan). Amplification was performed using SYBR Premix Ex TaqTM (TaKaRa, Japan) in a 7500 Fast Real-Time PCR System. The cDNA was amplified using primer sets for *Adar1p150* (sense: 5'-TT CAAGGAAACGAAAGTGAACTCTGGG-3' and anti-sense: 5'-TGTGGGTCCCCTGAACCCTT-3'), *Adar1p110* (sense: 5'-CCATTGATTCCTGACTGAAGGTGGAA-3' and antisense: 5'-CGATTCCTCGGACGCTGCC-3'), *Adar2* (Sense: 5'-GTTTCGACAGGGACGAAGTGT-3' and antisense: 5'-TGGCGTCATACCCTCTAGCA-3'), *Adar3* (sense: 5'-ATATTCGTGCGGTTAAAAGAAGGTG-3' and antisense: 5'-ATCTCGTAGGGAGAGTGGA GTCTT G-3') and *Gapdh* (sense: 5'-AGGTCGGTGTGAACGGATTTG-3' and antisense: 5'-TGTAGACCATGT AGTTGA GGTCA-3'). The PCR product was detected by 2% agarose gel electrophoresis.

### RNA sequencing and AEI analysis

Total RNAs extracted by Trizol reagent from the hippocampal tissues were quantified and identified by NanoDrop spectrophotometry (Thermo Scientific, USA). RNA was purified by RNasey Mini Kit (QIAGEN, Germany), and checked for integrity by RNA 6000 Pico kit (Agilent, USA). The cDNA libraries were constructed by using TruSeq Stranded mRNA Library Prep Kit (Illumina, USA) according to the protocol of manufacturer. Sequencing library was determined by using a HiSeq XTen platform (Illumina USA), and paired-end reads were generated, then followed by cluster generation. Quality control was performed on raw reads using fastqc. The raw reads were combined using multiqc with aligned reads (Ewels et al. 2016). The aligned reads analysis was performed with R using DESeq2 (Love et al. 2014) with standard parameters to generate differential gene expressions. The significant differential expression was defined by adjusted P value < 0.05 and absolute log2[fold change] > 1. Gene ontology analysis was carried out by String online tools (https:// string-db.org). To assess the A-to-I RNA editing of ADARs, the Alu Editing Index (AEI) was calculated as previously described (Roth et al. 2019). The variant index or AEI is defined as the weighted average of the A-to-G or any mismatches (between the reference genomic sequence and RNA-seq reads aligned to the regions of interest) to the total number of adenosines in Alu elements (or other nucleotide of interest) across the transcriptome within the brain region. The AEI tool is available at https://github.com/a2iEditing/RNAEditingIndexer.

### Behavior study

After calculation of sample size with GPower 3.1 software, a total of 64 male rat pups (n = 16 in each group, PND 7) were treated with control gas mixture (50% N_2_ plus with 50% O_2_, 6 h), sevoflurane (3%, 6 h), cadRNAs plus sevoflurane (3%, 6 h), or cadRNAs alone. Open field test was conducted to measure the possible locomotor activity deficits. Each animal was released in the center of the arena (100 cm × 100 cm × 40 cm). In order to avoid the presence of olfactory cues, the arena was carefully cleaned after each test. Activity was determined as the total distance traveled in 10 min.

The spatial cognitive functions of animals were examined by Morris water maze (MWM) as previously described. In training phase, all animals were allowed to learn the spatial relationship between the escape platform (submerged 2.0 cm, not visible) and distant cues. All animals received four trails each day for consecutive four days. Each rat was allowed 1 min to find the platform upon which they were allowed to stay for 20 s. If a rat did not reach the platform within 1 min, the rat was guided to the platform and stay there for 20 s. Latency was defined as the time to reach the platform. Probe test was carried out to examine the capability of memory retention. In probe test, single trial was performed after removing the original platform. Each animal was started to swim facing the pool wall in a random quadrant. All animals were allowed to swim for 1 min. The swimming time spent in each quadrant was analyzed. The number of crossings over the place where the platform used to be was recorded.

The fear conditioning experiment was carried out to assess the emotional cognitive functions as previously described (Wang et al. 2016). Briefly, the animal was allowed to freely explore for 5 min in a conditioning chamber. Then three unconditioned stimulus (1 mA foot shock for 1 s) and conditioned stimulus (80 db noise for 20 s) pairings were delivered and separated by 1 min each. Unconditioned stimulus was delivered after the conditioned stimulus presentation. At 24 h after conditioning, the contextual test was conducted in the absence of noise for 5 min in the same conditioning chamber. At 48 h after conditioning, the cued test was carried out by presentation of a cue (80 db noise) for 3 min with distinct visual and tactile cues in an alternative context. Freezing behavior was defined as the absence of all visible movement except for respiration.

### Statistical analysis

Statistical analyses were performed and constructed by GraphPad Prism 8.0 software. Statistical significance between two datasets was analyzed using two tailed Student’s t test. One-way ANOVA was performed to compare more than two groups followed by Tukey multiple comparison testing. For data of place trials in MWM, the repeated two-way measures ANOVA was employed for analysis followed by Bonferroni correction for multiple comparison testing. All data are expressed as mean ± SEM. In all experiments, statistical significance was set at P < 0.05 (* P < 0.05, **P < 0.01, ***P < 0.001).

## Results

### *Sevoflurane priming leads to hippocampal neuronal death *in vitro* and transcriptional alternation *in vivo

**Fig. 1:**
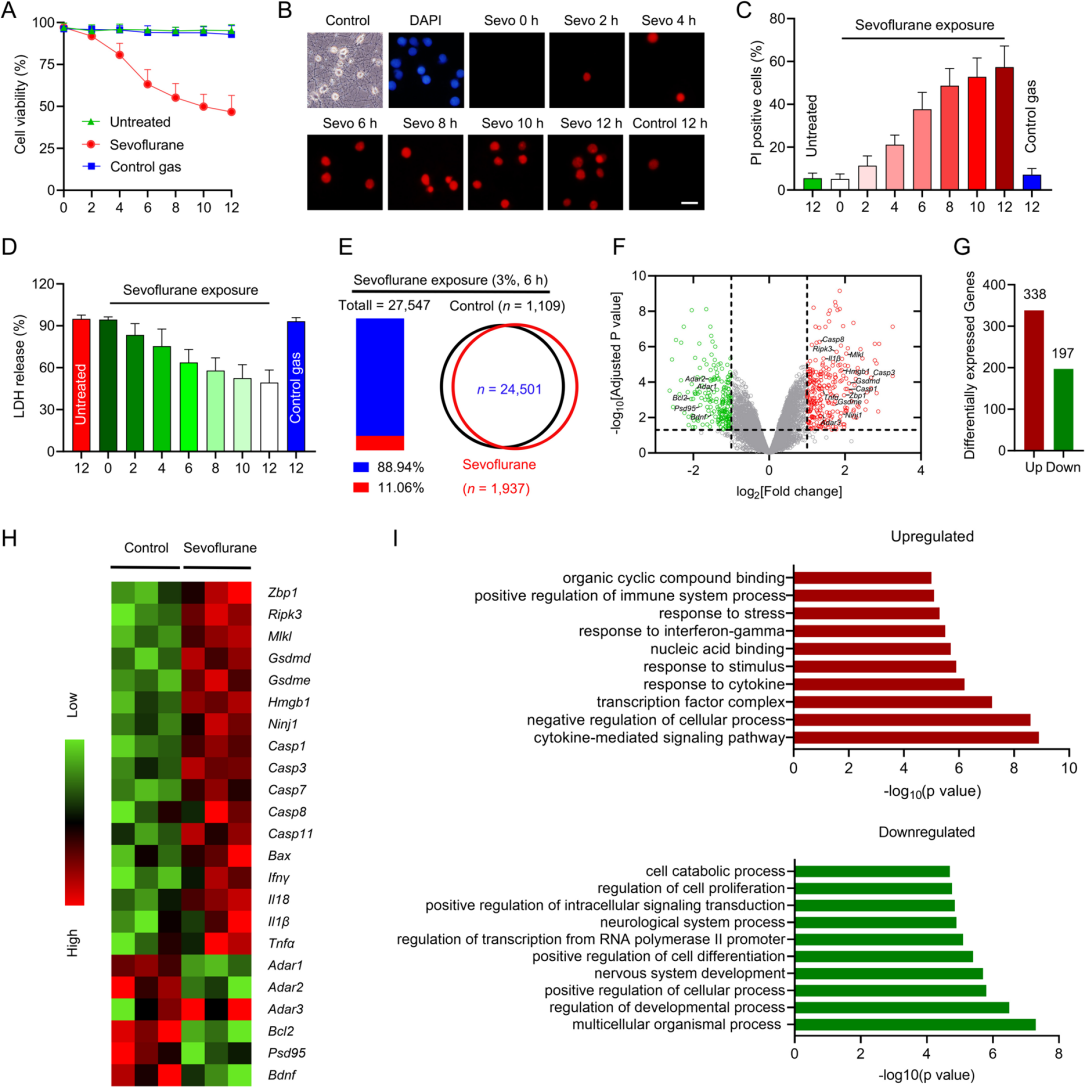
Sevoflurane priming leads to neuronal death and transcriptional alternation in the developing brain. **A** The hippocampal neurons were primed with 3% of sevoflurane for 2, 4, 6, 8, 10 or 12 h. The neuronal cultures of control group were flushed with gas mixture (95% O_2_ and 5% CO_2_) without sevoflurane for different hours as indicated. The cell viability was then evaluated by ToxiLight cytotoxicity bioassay. **B** After treatment with sevoflurane or control gas (95% O_2_ and 5% CO_2_) as indicated, PI-staining was performed. Scale bar = 10 μm. **C** The histogram represents the quantification of PI-positive cells. **D** The neuronal cultures were primed as indicated, the LDH release was assessed to determine cell death. **E** The hippocampal tissues of rap pups were subjected to RNA-seq following sevoflurane exposure (3%, 6 h). The Venn diagram illustrates gene transcripts that were detected in both groups. **F** Volcano plot depicts the distribution of differential expressed genes detected by RNA sequencing of hippocampal tissues derived from sevoflurane exposed rat pups. **G** The histogram represents the different expressed genes in the presence of sevoflurane. **H** The heatmap illustrates the significant different expressed genes involved in this study. **I** Gene ontology analysis of different expressed genes of hippocampal tissues from rat pups in sevoflurane neurotoxicity

To assess sevoflurane neurotoxicity, neuronal cultures (DIV 7) were exposed to 3% sevoflurane for 2, 4, 6, 8, 10 or 12 h. After sevoflurane treatment, neuronal cultures were subjected to evaluation of cell viability by ToxiLight assay. We found that 3% of sevoflurane time-dependently diminished cell viability compared with the control group (Fig. 1A), confirming by analysis of PI positive cells (Fig. 1B-C). To validate sevoflurane neurotoxicity, cell death was determined by the release of the cytosolic enzyme LDH into the extracellular space, because LDH is only released from post-lytic cells (Evavold et al. 2021). Consistently, time-dependent cell death was also observed by analysis of LDH release (Fig. 1D). In contrast, treatment with control gas mixture (95% air and 5% CO_2_) for 12 h had no effect on cell viability and cell death (Fig. 1A-D), implying that sevoflurane itself is responsible for the neuronal loss. As 3% sevoflurane exposed for 6 h is sufficient to lead to anesthetic neurotoxicity, this treatment paradigm was adopted in the following experiments.

To transcriptionally examine developmental sevoflurane neurotoxicity, rat pups (PND 7) were subjected to sevoflurane exposure (3%, 6 h), and the hippocampal tissues were harvested for RNA sequencing (RNA seq). Analysis of RNA seq showed that 88.94% of transcripts were detected in control and sevoflurane treated samples (Fig. 1E), and volcano plot of RNA seq was shown in Fig. 1F. Analysis of differentially expressed genes showed that 338 genes were up-regulated, while 197 genes were down-regulated after sevoflurane exposure (Fig. 1G). Further examination showed that *Gsdmd*, *Gsdme*, *Caspases*, *Ripk3*, *Mlkl* and inflammatory factors (e.g. *Il18*, *Il1β*, *Tnf*α and *Ifnγ*) were increased by sevoflurane priming (Fig. 1H), intimating that PANoptosis and inflammatory response may be involved in this process. Gene ontology analysis showed that cytokine-mediated signaling pathway, negative regulation of cellular process, and response to cytokine or stimulus were promoted by sevoflurane treatment (Fig. 1I). In contrast, regulation of developmental process, nervous system development, neurological system process and positive regulation of cell differentiation were inhibited by sevoflurane exposure (Fig. 1I). These observations indicate that sevoflurane exposure leads to hippocampal neuronal death in vitro and transcriptional alternation in vivo.

### Sevoflurane exposure triggers PANoptosis and elicits inflammatory response in the developing brain

**Fig. 2:**
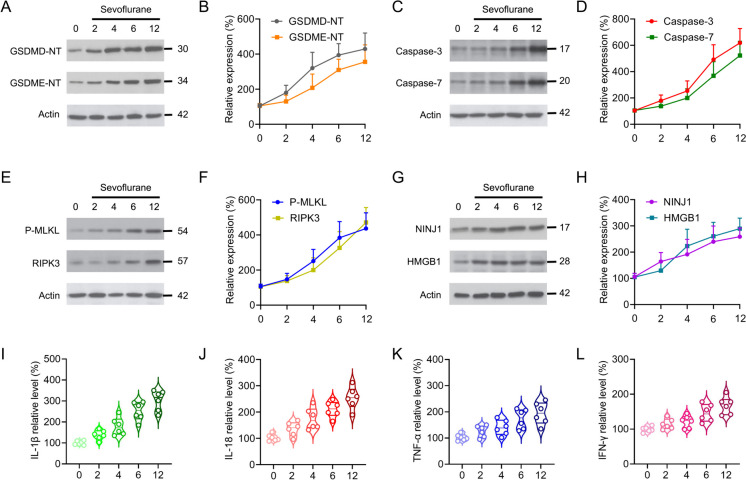
Sevoflurane exposure triggers PANoptosis and inflammatory response in the developing brain. **A-H** The primary hippocampal neurons were treated with 3% of sevoflurane for 2, 4, 6 or 12 h as indicated. Neuronal cultures were collected for detecting the expressions of GSDMD-NT, GSDME-NT, cleaved Caspase-3, cleaved Caspase-7, phosphorylated MLKL (P-MLKL), RIPK3, NINJ1 and HMGB1 by immunoblot. The Actin and voltage-dependent anion channel 1 (VDAC1) were detected as plasma and membrane loading control, respectively. The line charts represent the quantification of relative expressions of GSDMD-NT, GSDME-NT, cleaved Caspase-3, cleaved Caspase-7, P-MLKL, RIPK3 NINJ1 and HMGB1. **I-L** After exposed to 3% of sevoflurane for 2, 4, 6 or 12 h, the neuronal cultures were harvest for assessing the relative levels of IL-1β, IL-18, TNF-α and IFN-γ by ELISA

To identify neuronal pyroptosis, we examined the cleavage of the pyroptotic effector gasdermin D N-terminal (GSDMD-NT) and GSDME-NT. Sevoflurane exposure led to production of the active GSDMD-NT P30 and GSDME-NT P34 fragments (Fig. 2A-B). In addition, sevoflurane also triggered the activation of apoptotic effectors, as evidenced by the cleavage of apoptotic Caspase-3 and Caspase-7 (Fig. 2C-D). Next, we monitored necroptotic effector RIPK3 and phosphorylated MLKL (P-MLKL) in developmental sevoflurane neurotoxicity. An elevated level of RIPK3 and P-MLKL were detected after sevoflurane priming (Fig. 2E-F), indicating that the activation of necroptotic effectors is occurring. Ninjurin-1 (NINJ1) is required for plasma membrane rupture and subsequent release of High-mobility group box 1 (HMGB1) and LDH (Kayagaki et al. 2021). Further investigation showed that both NINJ1 and HMGB1 were significantly increased in the scenario of sevoflurane (Fig. 2G-H), indicating that neuronal membrane rupture is implicated in this process. Moreover, analysis of the inflammatory profile of sevoflurane neurotoxicity showed that the levels of IL-1β, IL-18, TNF-α and IFN-γ were enhanced pronouncedly in a time-dependent manner (Fig. 2I-L). Collectively, these data suggest that sevoflurane treatment sensitizes the neurons to undergo cell death and inflammasome activation involving the components of PANoptosis.

### ADAR1-P150, but not ADAR1-P110, attenuates neuronal death in developmental sevoflurane neurotoxicity

**Fig. 3:**
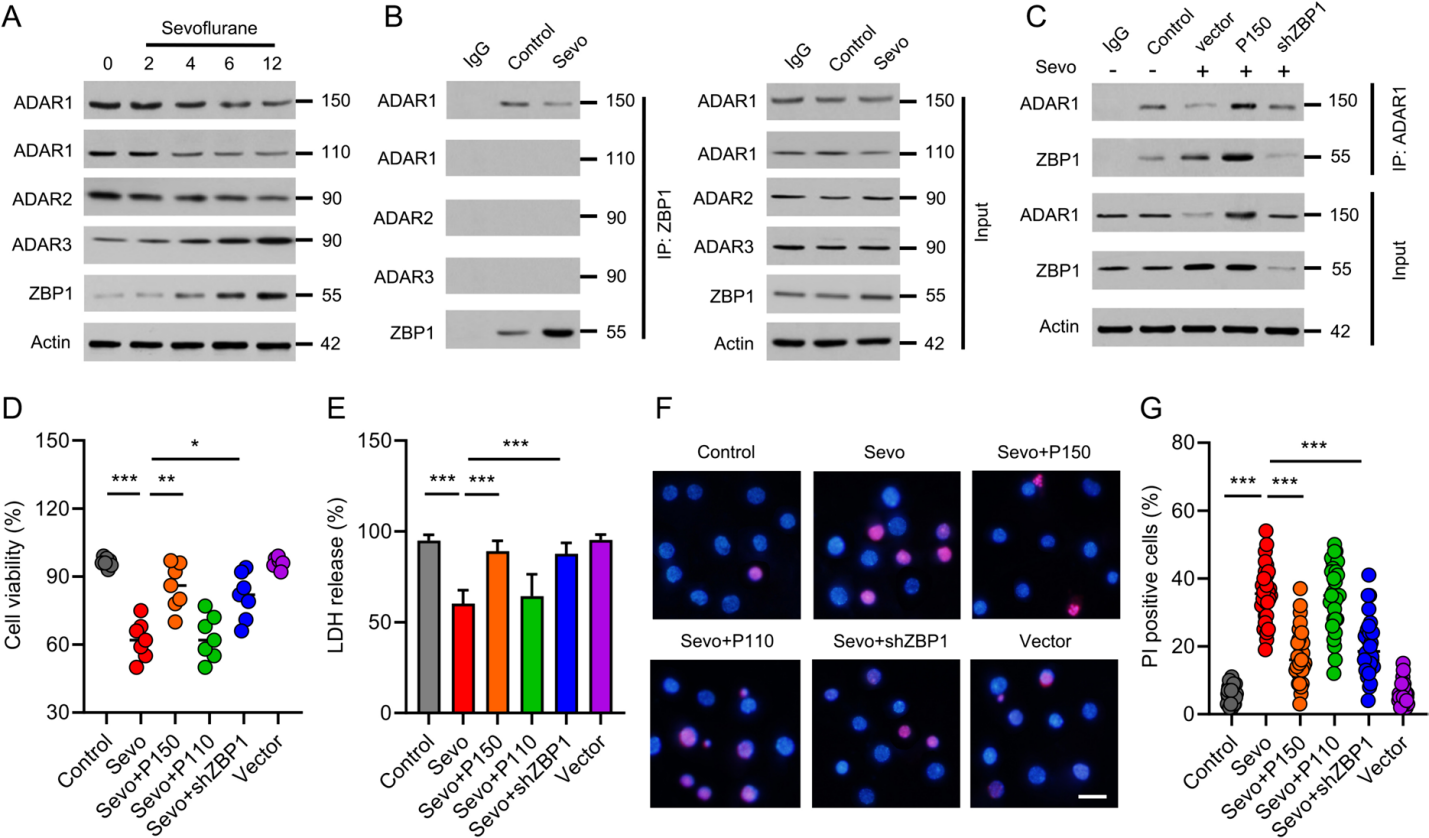
ADAR1-P150, but not ADAR1-P110, attenuates neuronal death in sevoflurane neurotoxicity. **A** The primary hippocampal neurons were treated with 3% of sevoflurane for 2, 4, 6 or 12 h. After sevoflurane exposure, the cells were collected for evaluating the expressions of ADAR1-P150, ADAR1-P110, ADAR2, ADAR3 and ZBP1 by immunoblot. Actin was probed as a loading control. **B** The neuronal cultures were primed with sevoflurane (3%, 6 h), and then lysed for ZBP1 immunoprecipitation followed by probing the possible interaction with ADAR1-P150, ADAR1-P110, ADAR2 or ADAR3 via immunoblot. Actin was detected as a loading control. **C** Hippocampal neurons were transfected with pmGFP-ADAR1-P150 to enhance ADAR1-P150 expression. ZBP1 shRNA was transfected to knock down ZBP1 expression. After sevoflurane priming (3%, 6 h), the neurons were lysed for ADAR1 immunoprecipitation followed by probing the possible interaction with ZBP1 by immunoblot. **D** The neuronal cultures were transfected with pmGFP-ADAR1-P150, pmGFP-ADAR1-P110 or ZBP1 shRNA before sevoflurane treatment. The cell viability was assessed by ToxiLight cytotoxicity bioassay. **E** The neuronal cultures were treated as indicated. LDH release was detected to determine cell death. **F** The neuronal cultures were treated as indicated. PI-staining was performed after treated as indicated. Scale bar = 10 μm. **G** The percentage of PI positive cells was analyzed. Data are shown as mean ± SEM. *P < 0.05, **P < 0.01, ***P < 0.001

ADAR1 and ZBP1 are the only two proteins with Zα domain in mammals. Since ZBP1 was activated by sevoflurane treatment, we had assumed that ADAR1 would also be activated by sevoflurane exposure. Unexpectedly, the protein expressions of ADAR1-P150, ADAR1-P110 and ADAR2 were inhibited, while ADAR3 was enhanced, by sevoflurane treatment (Fig. 3A). In order to directly examine the connection between ADAR1 and ZBP1, Co-IP was performed in the presence of sevoflurane. We found that ADAR1-P150, but not ADAR1-P110, ADAR2 or ADAR3, was pulled down by ZBP1 antibody (Fig. 3B). Conversely, ZBP1 could also be pulled down by ADAR1-P150 antibody in the same condition (Fig. 3C). To interrogate the role of ADAR1-ZBP1 signaling in developmental sevoflurane neurotoxicity, we transfected pmGFP-ADAR1-P150 plasmid to enhance ADAR1-P150 expression, and ZBP1 shRNA to depress ZBP1 expression in hippocampal neuronal cultures. We found that overexpression of ADAR1-P150 promoted, while knock-down of ZBP1 reduced, the interaction between ADAR1 and ZBP1 (Fig. 3C). To further examine the contributed role of ADAR1-P150 and ADAR1-P110 in sevoflurane neurotoxicity, the pmGFP-ADAR1-P150 or pmGFP-ADAR1-P110 plasmid was transfected before sevoflurane priming. Strikingly, we found that overexpression of ADAR1-P150, but not ADAR1-P110 significantly improved cell viability (Fig. 3D) and LDH release (Fig. 3E). These results were confirmed by analysis of PI positive cells (Fig. 3F-G). Overall, these evidences indicate that ADAR1-P150, but not ADAR1-P110, prevents neurotoxicity in developmental sevoflurane exposure.

### *ADAR1 P150 inhibits sevoflurane-induced ZBP1-dependent PANoptosis and inflammatory responses *in vivo

**Fig. 4:**
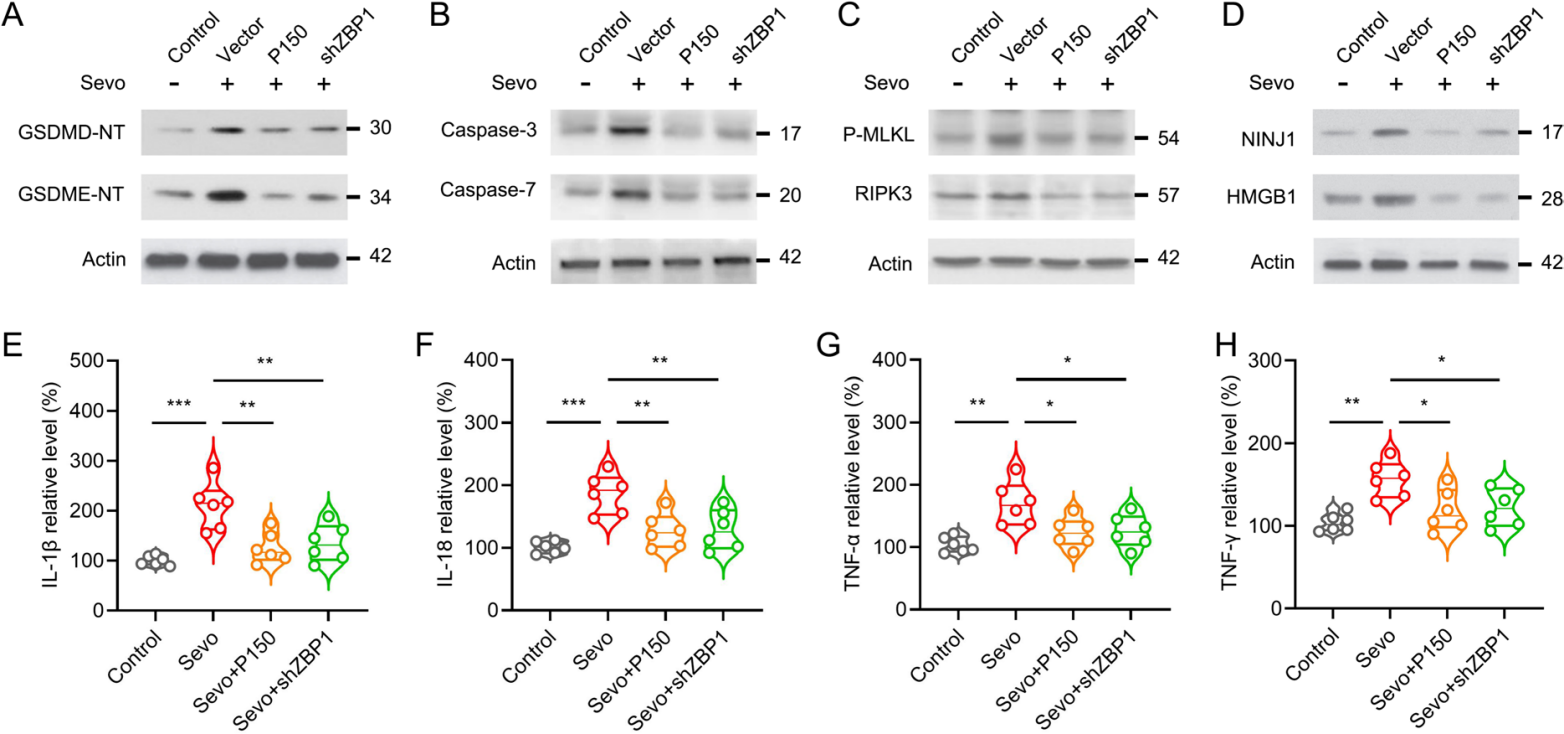
ADAR1 P150 inhibits ZBP1-dependent PANoptosis and inflammatory response in sevoflurane priming. **A-D** Hippocampal neurons were transfected with pmGFP-ADAR1-P150 to enhance ADAR1-P150 expression. ZBP1 shRNA was transfected to knock down ZBP1 expression. After sevoflurane priming (3%, 6 h), the neuronal cultures were collected for detecting the expressions of GSDMD-NT, GSDME-NT, cleaved Caspase-3, cleaved Caspase-7, phosphorylated MLKL (P-MLKL), RIPK3, NINJ1 and HMGB1 by immunoblot. The Actin and voltage-dependent anion channel 1 (VDAC1) were detected as plasma and membrane loading control, respectively. **E–H** After treatment as indicated, the neuronal cultures were exposed to 3% of sevoflurane for 6 h. The neuronal cultures were harvest for assessing the relative levels of IL-1β, IL-18, TNF-α and IFN-γ by ELISA. Error bars indicate the mean ± SEM. *P < 0.05, **P < 0.01, ***P < 0.001

Since PANoptosis and inflammatory effectors were involved in sevoflurane neurotoxicity, we next sought to explore the role of ADAR1-ZBP1 signaling in this setting. Based on the above evidences, we assumed that overexpression of ADAR1-P150 or knock-down of ZBP1 would attenuate sevoflurane neurotoxicity in the developing brain. The pmGFP-ADAR1-P150 plasmid or ZBP1 shRNA was transfected in vivo before sevoflurane treatment. After treatmet with sevoflurane for (3%, 6 h), the hippocampal tissues of animals were collected to examine the effectors of PANoptosis. We found that ADAR1-P150 overexpression or ZBP1 knock-down significantly inhibited the expressions of pyroptotic (GSDMD-NT and GSDME-NT), apoptotic (cleaved Caspase-3 and cleaved Caspase-7) and necroptotic (P-MLKL and RIPK3) effectors (Fig. 4A-C). In addition, ADAR1-P150 overexpression or ZBP1 knock-down also pronouncedly restricted the expressions of NINJ1 and HMGB1 (Fig. 4D), which are marked molecules of plasma membrane rupture. Moreover, sevoflurane-induced inflammatory factors (IL-1β, IL-18, TNF-α and IFN-γ) could also be restored by ADAR1-P150 overexpression or ZBP1 knock-down (Fig. 4E-H). Taken together, our experimental data support an essential role of ADAR1-P150 as a suppressor of ZBP1-dependent PANoptosis and inflammatory responses in developmental sevoflurane neurotoxicity.

### ADAR1-P150 competes with ZBP1 for binding to Z-RNA in developmental sevoflurane neurotoxicity

**Fig. 5:**
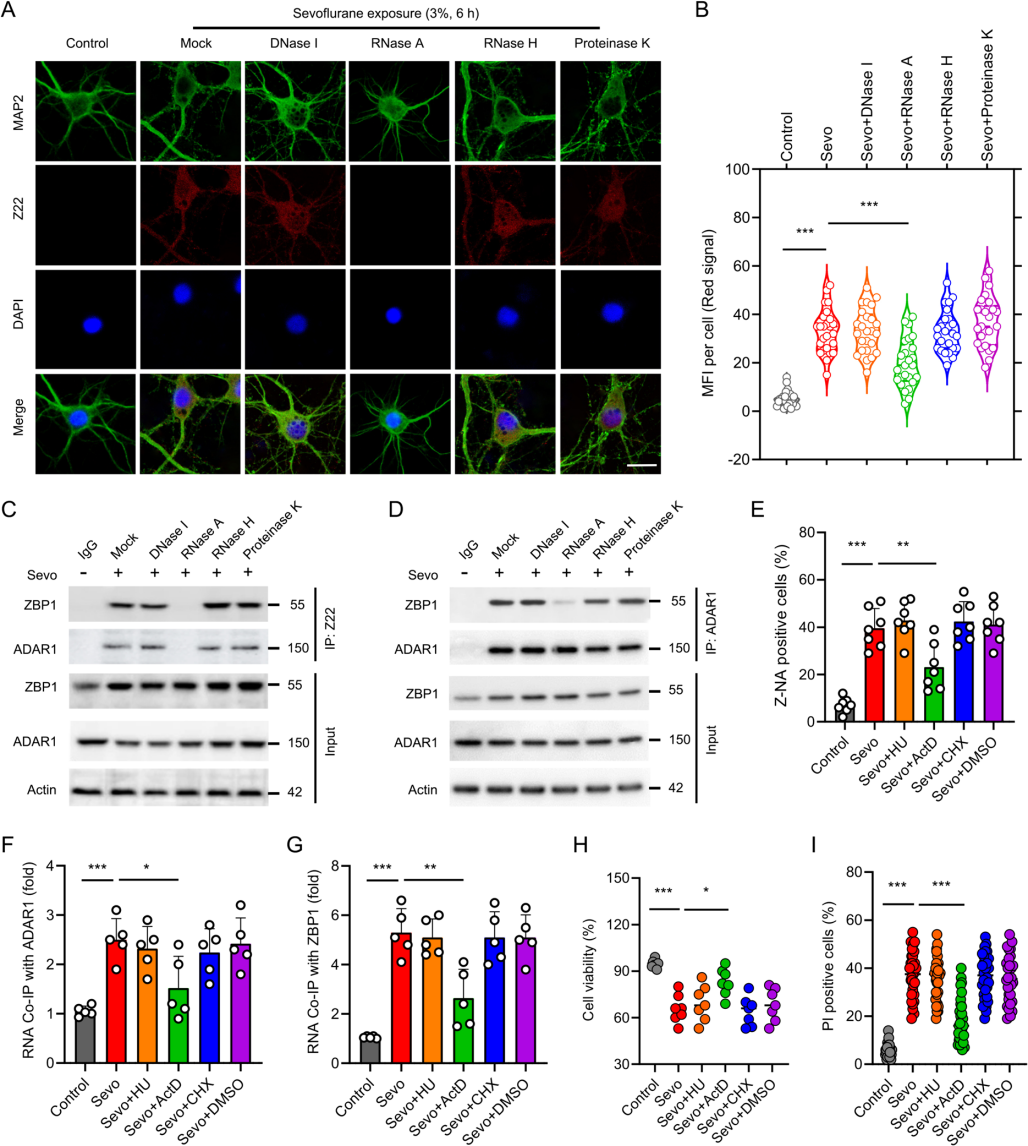
ADAR1-P150 competes with ZBP1 for binding to Z-RNA in developmental sevoflurane neurotoxicity. **A** The hippocampal neurons were primed with 3% sevoflurane for 6 h, followed by treatment with DNase I, RNase A, RNase H, Proteinase K or left untreated (mock). The neurons were then stained by MAP2 and Z22 antibody, and the nucleus was stained by DAPI. Scale bar = 10 μm. **B** Quantification of the median fluorescence intensity of Z-RNA staining by Z22 antibody. For each group, 30 individual cells were analyzed. **C** The neuronal cultures were treated as indicated, and were then lysed for Z22 immunoprecipitation followed by probing the possible interaction with ZBP1 or ADAR1 by immunoblot. **D** The neuronal cultures were treated as indicated, and were then lysed for ADAR1 immunoprecipitation followed by probing the interaction with ZBP1 by immunoblot. **E** The cells were pretreated with indicated inhibitors of DNA synthesis (hydroxyurea, HU, 10 mM), RNA transcription (actinomycin D, Act D, 2 mg/mL) or protein synthesis (cycloheximide, CHX, 40 mg/mL). After exposed to sevoflurane (3%, 6 h), the cells were stained by Z22 antibody followed by flow cytometry analysis. The histogram represents the quantification of Z-NA positive cells. **F-G** Co-immunoprecipitation (Co-IP) of RNA bound to ADAR1 or ZBP1. The neuronal cultures were treated as indicated, followed by UV crosslink and lysis. Either ADAR1- or ZBP1-associated RNA was isolated by Co-IP with anti-ADAR1 antibody or anti-ZBP1 antibody. RNA associates with either of these immunoprecipitates were isolated by Trizol. The RNAs were then quantified by NanoDrop. **H** The cells were treated as indicated, and cell viability was evaluated by ToxiLight cytotoxicity bioassay. **I** PI positive cells were analyzed for each group. Error bars indicate the mean ± SEM. Statistical significance was set at P < 0.05 (* P < 0.05, **P < 0.01, ***P < 0.001)

ZBP1 was reported to sense Z-RNAs generated by replication of influenza A virus, initiating inflammatory response and cell death (Zhang et al. 2020). Since both ADAR1-P150 and ZBP1 contain a Zα domain that can bind Z-NAs (Z-DNA and Z-RNA), we then reasoned that ADAR1-P150 may compete with ZBP1 for binding to Z-RNA in sevoflurane neurotoxicity. To test this possibility, we first examine the presence of Z-RNA using Z22 clone antibody, which was originally raised against Z-DNA. Recently, Z22 clone was also shown to characterize Z-RNA (Koehler et al. 2021). We found that the mean fluorescence intensity was significantly greater in the model of developmental sevoflurane neurotoxicity (Fig. 5A-B), suggesting the accumulation of Z-NAs. Importantly, the Z22 clone produced signal was also sensitive to RNase A but not to DNase I, RNase H or Proteinase K (Fig. 5A-B), indicating that it originated from the accumulation of Z-RNAs rather than Z-DNAs or from DNA:RNA hybrids. To confirm this observation, we collected sevoflurane treated neuronal cultures and immunoprecipitated the cell lysate with Z22 antibody. As expected, both ADAR1-P150 and ZBP1 could be pulled down by Z22 antibody (Fig. 5C). This interaction was abolished when Z22 antibody:bead complexes were treated with RNase A, but resisted to treatment with DNase I, RNase H or Proteinase K (Fig. 5C).

To elucidate the competition of ADAR1-P150 with ZBP1 for binding to Z-RNA, the sevoflurane exposed cell lysates were pulled down by ADAR1-P150. The immunoprecipitated ZBP1 was significantly reduced by treatment with RNase A, but not DNase I, RNase H or Proteinase K (Fig. 5D). To interrogate the bridge role of Z-RNA in association between ADAR1-P150 and ZBP1, we co-incubated hippocampal neurons with the inhibitors of DNA synthesis (hydroxyurea, 10 mM), RNA transcription (actinomycin D, 2 mg/mL) or protein synthesis (cycloheximide, 40 mg/mL) during sevoflurane administration. Flow cytometry analysis showed that sevoflurane priming dramatically promoted Z-NA positive cells, which could be abrogated by treatment with actinomycin D rather than hydroxyurea or cycloheximide (Fig. 5E). In addition, we also evaluated mRNA levels by using RIP assay. After ADAR1-P150 or ZBP1 associated RNA was isolated by immunoprecipitation with anti-ADAR1-P150 or anti-ZBP1 antibody respectively, RNA was then purified and quantified by NanoDrop. We found that RNA Co-immunoprecipitated with anti-ADAR1-P150 or anti-ZBP1 antibody was significantly boosted by sevoflurane priming. Treatment with actinomycin D, but not hydroxyurea or cycloheximide, significantly alleviated the RNA immunoprecipitated with anti-ADAR1-P150 or anti-ZBP1 antibody (Fig. 5F-G), consistent with a Z-RNA bridge between ADAR1-P150 and ZBP1. Furthermore, actinomycin D co-incubation with sevoflurane significantly improved cell viability (Fig. 5H) and PI positive cells (Fig. 5I).

### ADAR1 A-to-I RNA editing improves developmental sevoflurane-induced cell death and PANoptosis

**Fig. 6:**
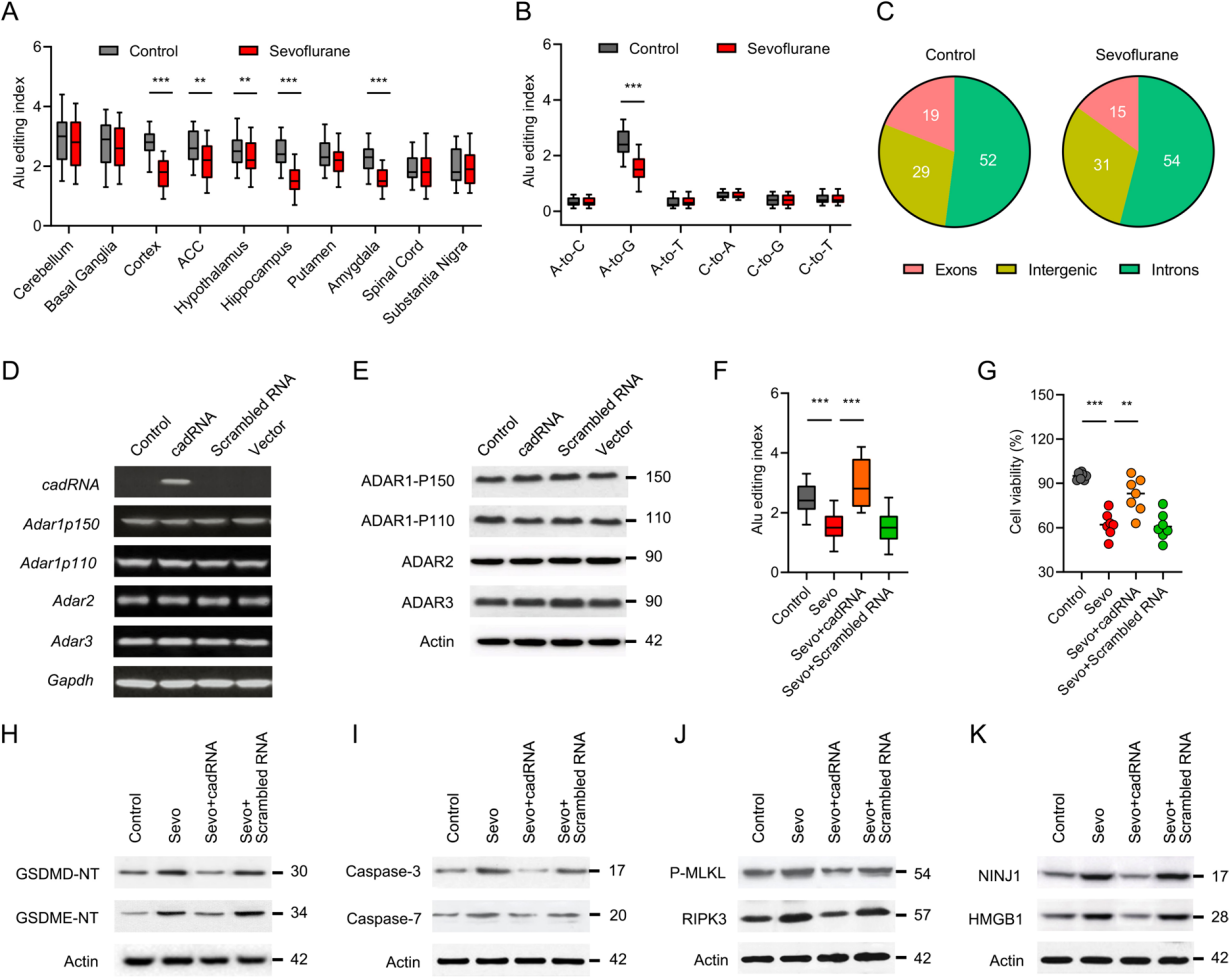
ADAR1 A-to-I RNA editing improves developmental sevoflurane-induced cell death and PANoptosis. **A** Rat pups (PND 7) were exposed to sevoflurane (3%, 6 h), and the different brain regions were collected for Alu editing index (AEI) analysis. Distributions are presented as box-and-whisker plots (horizontal lines, medians; box edges, upper and lower quartiles; whiskers, the interquartile range), depicting the Alu editing index values of different brain regions (ACC, Anterior Cingulate Cortex) in the presence of sevoflurane. **B** AEI of hippocampal region was analyzed after sevoflurane priming. Adenosine to guanosine (A-to-G) represents A-to-I editing, because the sequencing machinery detects inosines as guanosines. **C** Pie chart represents the distribution of mismatched editing sites (Mismatches, %) after sevoflurane treatment. **D** The neuronal cultures were transfected with cadRNAs, Scrambled RNA or vector, an equal number of neurons were analyzed by PCR for *cadRNAs*, *Adar1p150*, *Adar1p110*, *Adar2* and *Adar3*. *Gapdh* gene was analyzed as a loading control.** E** After treated as indicated, cell lysates were probed by immunoblot for the expressions of ADAR1-P150, ADAR1-P110, ADAR2 and ADAR3. Actin was reprobed as a loading control. **F** Rat pups were retro-orbitally injected with AAV-delivered cadRNAs followed by sevoflurane exposure (3%, 6 h) 48 h later. Alu editing index values were calculated for each group. **G** After treatment as indicated, cell viability was assessed by ToxiLight cytotoxicity bioassay. **H–K** The hippocampal neurons were transfected with cadRNAs, Scrambled RNA or vector followed by sevoflurane priming (3%, 6 h) 48 h later. The neuronal cultures were analyzed by immunoblot for the relative expressions of GSDMD-NT, GSDME-NT, cleaved Caspase-3, cleaved Caspase-7, Phosphorylated MLKL (P-MLKL), RIPK3 NINJ1 and HMGB1. The Actin and voltage-dependent anion channel 1 (VDAC1) were detected as plasma and membrane loading control, respectively. Error bars indicate the mean ± SEM. Statistical significance was determined at **P < 0.01, ***P < 0.001

We have demonstrated that sevoflurane exposure gives rise to inhibition of ADAR1 expression. Since one of the most important roles of ADAR1 is to edit RNA A-to-I modification, we reasoned that ADAR1 may prevent ZBP1-dependent PANoptosis via A-to-I RNA editing in sevoflurane neurotoxicity. To better understand the effects of sevoflurane exposure on RNA editing in different brain regions, we applied an AEI as a global measure of brain region-specific RNA editing activity. The AEI is calculated as the total number of A to G mismatches divided by the coverage of adenosines. We found that sevoflurane exposure significantly decreases AEI value in specific brain regions, including hippocampus, amygdala, cortex, hypothalamus and anterior cingulate cortex (Fig. 6A). These brain regions coincided well with the neurodegenerative region induced by neonatal exposure to sevoflurane (Satomoto et al. 2009). Strikingly, no significant difference in the indices of other common mismatches, except for A-to-G mismatch, or RNA editing sites catalyzed by cytidine deaminases was observed in hippocampus (Fig. 6B). In line with the previous report (Roth et al. 2019), the involved editing activity predominantly resides in intergenic and introns regions in a promiscuous fashion, while a handful of sites resides in coding regions (Fig. 6C).

Since the Alu sequences are primarily edited by ADAR1 (Bahn et al. 2015), we had postulated that rectification of RNA editing may ameliorate sevoflurane neurotoxicity. To validate this hypothesis, we then utilized AAV to deliver engineered circular ADAR-recruiting guide RNAs (cadRNAs) into cells, which is capable of recruiting endogenous adenosine deaminases to promote cellular A-to-I RNA editing. The cadRNAs are highly specific, with limited editing of non-target adenosines in the target region and rare global off-targets (Yi et al. 2022). We demonstrated that the AAV-delivered cadRNAs did not significantly alter the mRNA (Fig. 6D) and protein (Fig. 6E) expressions of ADAR1-P150, ADAR1-P110, ADAR2 and ADAR3 as compared with scrambled RNAs, which are also specific but have no functions of A-to-I RNA editing. However, the presence of cadRNAs substantially increased the AEI value of hippocampal region (Fig. 6F). In addition, exogenous cadRNAs promoted the cell viability induced by sevoflurane exposure, while scrambled RNAs failed to improve cell viability (Fig. 6G). Next, we investigated the anti-PANoptosis role of cadRNAs in sevoflurane neurotoxicity. We found that cadRNAs transfection significantly regressed the effectors of pyroptosis (GSDMD-NT and GSDME-NT, Fig. 6H), apoptosis (cleaved Caspase-3 and cleaved Caspase-7, Fig. 6I) and necroptosis (P-MLKL and RIPK3, Fig. 6J). Moreover, delivery of cadRNAs also relieved the cell rupture associated factors NINJ1 and HMGB1 (Fig. 6K). Collectively, these results indicated that enhancing A-to-I RNA editing by exogenous cadRNAs delivery prevents developmental sevoflurane-induced cell death and PANoptosis.

### ADAR1 A-to-I RNA editing inhibits Z-RNA production in developmental sevoflurane neurotoxicity

**Fig. 7:**
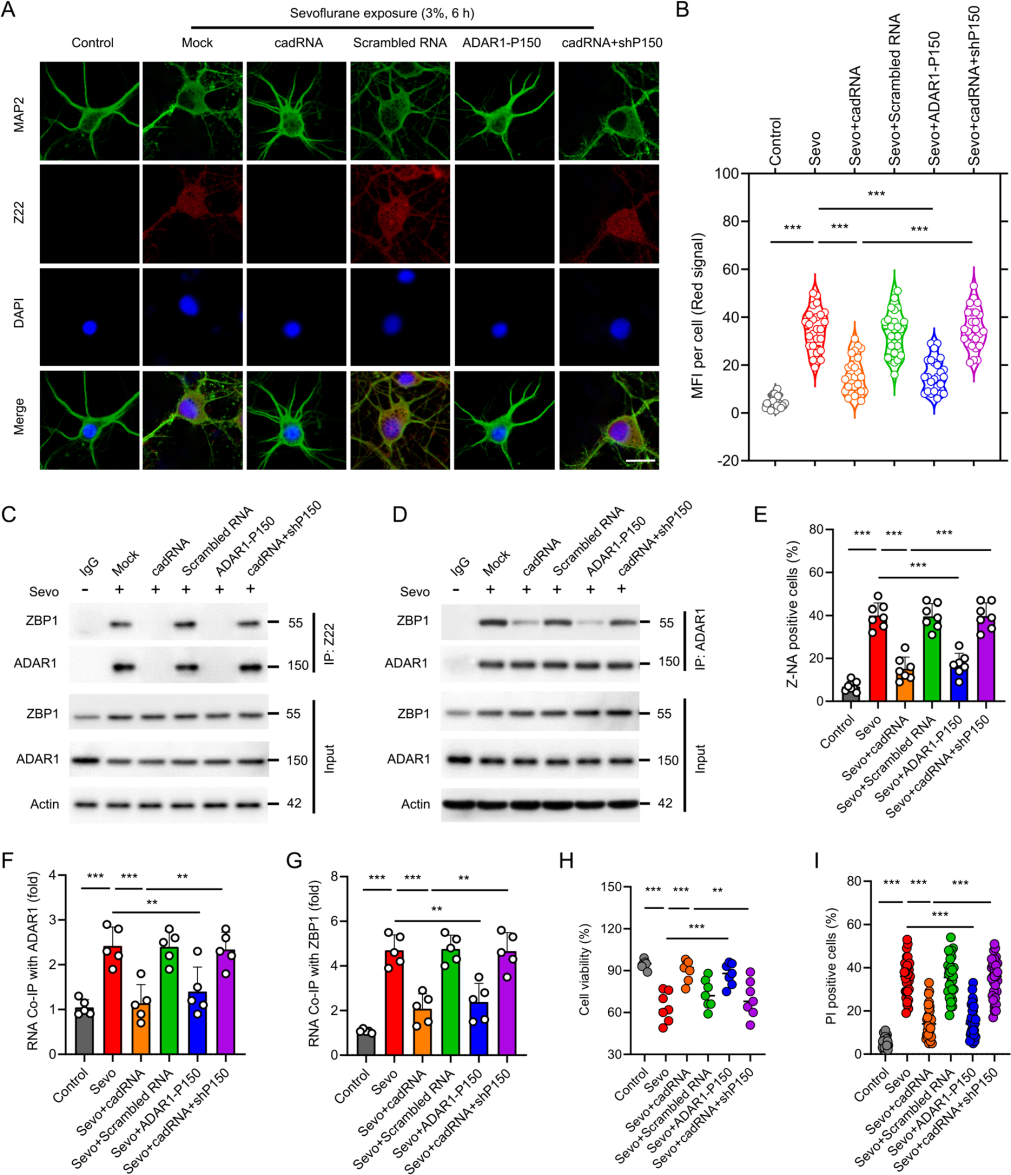
ADAR1 A-to-I RNA editing inhibits Z-RNA production in developmental sevoflurane neurotoxicity. **A** The hippocampal neurons were transfected with cadRNAs, Scrambled RNA, pmGFP-ADAR1-P150 or cadRNAs combined with ADAR1-P150 shRNA (shP150) for 48 h. The neurons were primed with 3% sevoflurane for 6 h, followed by staining with anti-MAP2 and Z22 antibodies, and the nucleus was stained by DAPI. Scale bar = 10 μm. **B** Quantification of the median fluorescence intensity of Z-RNA staining by Z22 antibody. For each group, 30 individual cells were analyzed. **C** The neuronal cultures were treated as indicated, and were then lysed for Z22 immunoprecipitation followed by probing the possible interaction with ZBP1 or ADAR1 by immunoblot. **D** The neuronal cultures were treated as indicated, and were then lysed for ADAR1 immunoprecipitation followed by probing the interaction with ZBP1 by immunoblot. **E** The cells were treated as indicated before sevoflurane exposure (3%, 6 h). The cells were stained by Z22 antibody followed by flow cytometry analysis. The histogram represents the quantification of Z-NA positive cells. **F-G** Co-immunoprecipitation (Co-IP) of RNA bound to ADAR1 or ZBP1. The neuronal cultures were treated as indicated, followed by UV crosslink and lysis. Either ADAR1- or ZBP1-associated RNA was isolated by Co-IP with anti-ADAR1 antibody or anti-ZBP1 antibody. RNA associates with either of these immunoprecipitates were isolated by Trizol. The RNAs were then quantified by NanoDrop. **H-I** The cells were treated as indicated, and cell viability was evaluated by ToxiLight cytotoxicity bioassay. The cell death was examined by analyzing the percentage of PI positive cells. Error bars indicate the mean ± SEM. Statistical significance was set at P < 0.05 (* P < 0.05, **P < 0.01, ***P < 0.001)

In order to decipher the contribution of A-to-I RNA editing of ADAR1-P150 to cellular Z-RNA production, we overexpressed ADAR1-P150 or delivered exogenous cadRNAs into hippocampal neurons. We found that both ADAR1-P150 overexpression and cadRNAs delivery significantly repressed the cellular accumulation of Z-RNAs in the scenario of sevoflurane (Fig. 7A-B). To dissect the role of ADAR1-P150 in A-to-I RNA editing, we transfected ADAR1-P150 shRNA to knock down ADAR1-P150 in the presence of cadRNAs before sevoflurane treatment. In the absence of ADAR1-P150, as expected, cadRNAs could not disrupt the accumulation of Z-RNAs (Fig. 7A-B), suggesting that ADAR1-P150 is responsible for the elimination of cellular Z-RNAs. Additionally, cadRNAs delivery compromised the interaction between Z-RNAs and ZBP1 or ADAR1 (Fig. 7C-D). Furthermore, cadRNAs also decreased the Z-NA positive cells (Fig. 7E), as well as diminished the RNA immunoprecipitated with anti-ADAR1-P150 or anti-ZBP1 antibody (Fig. 7F-G). These results are reminiscent of the bridge role of Z-RNAs connecting ZBP1 and ADAR1. Finally, cell viability and PI positive cells were rectified by exogenous cadRNAs (Fig. 7H-I). Of note, these effects of cadRNAs could be compromised by ADAR1-P150 shRNA transfection, confirming that cadRNAs exert its A-to-I RNA editing via ADAR1-P150. These findings strongly indicated that ADAR1-P150 A-to-I RNA editing is responsible for Z-RNA production in sevoflurane neurotoxicity.

### ADAR1 A-to-I RNA editing ameliorates developmental sevoflurane-induced neurocognitive deficits

**Fig. 8:**
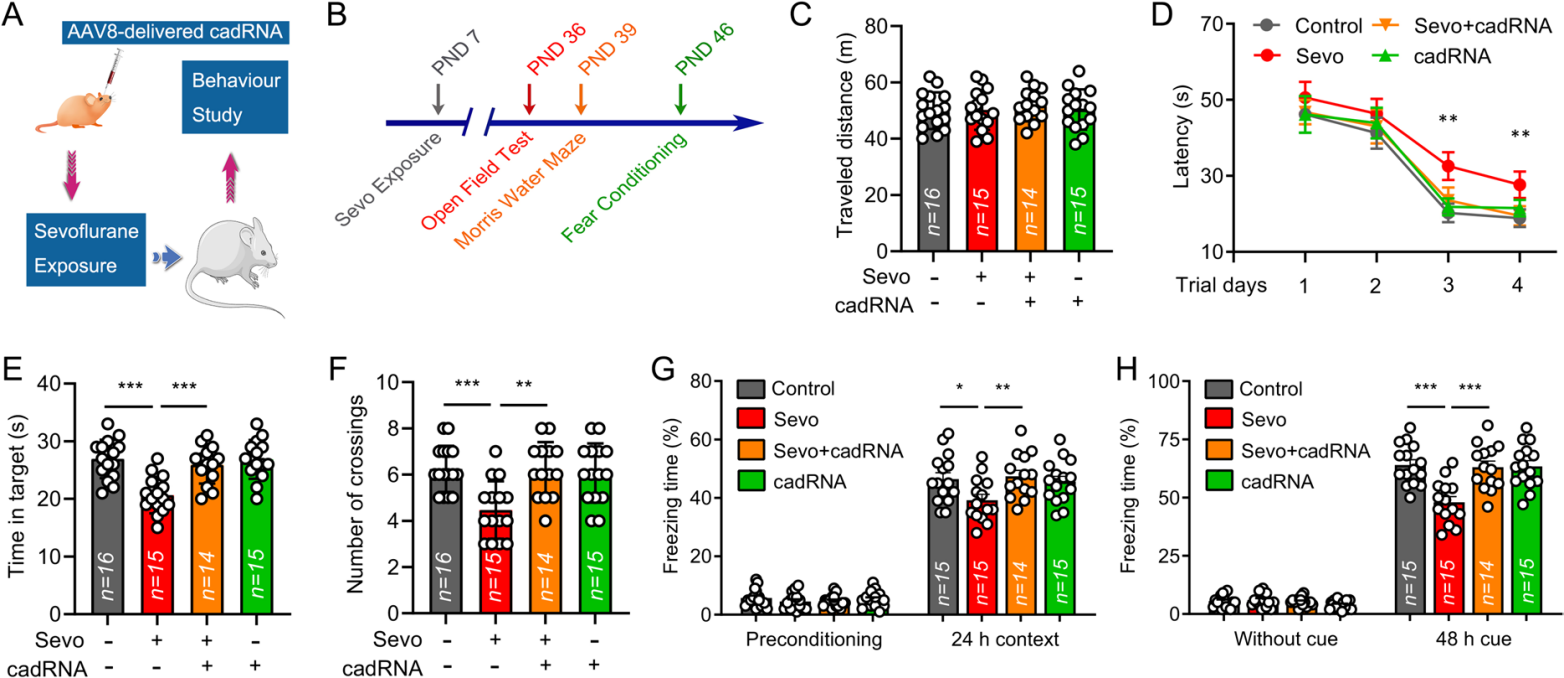
ADAR1 A-to-I RNA editing ameliorates developmental sevoflurane-induced neurocognitive deficits. **A** The schematic diagram shows the experimental procedures of behavior study. At postnatal day (PND) 5, rat pups were retro-orbitally injected with AAV-delivered cadRNAs followed by sevoflurane exposure (3%, 6 h) at PND 7. **B** The behavior study was performed 4 weeks after sevoflurane treatment. The Open field test, Morris water maze (MWM) and fear conditioning test were performed at PND 36, 39 and 46, respectively. **C** Open field test was performed to measure the possible locomotor activity impairments. The locomotor activity was determined as the total distance traveled in 10 min. **D** MWM was carried out to evaluate the capability of spatial cognitive functions. In place trials, latency was defined as the time to reach the submerged platform. **E** In probe test of MWM, the swimming track was analyzed. The histogram shows the time spend in targeted quadrant. **F** The histogram represents the number of crossings in probe test of MWM. **G** The fear conditioning test was performed to examine the effects of sevoflurane treatment on emotional cognitive functions. The histogram shows the percentage of freezing time 24 h after conditioning. **H** The histogram represents the percentage of freezing time in cued test 48 h after conditioning. Error bars indicate the mean ± SEM. Significance was determined at *P < 0.05, **P < 0.01

To assess the effects of ADAR1 A-to-I RNA editing on cognitive functions, AAV-delivered cadRNAs was injected retro-orbitally into rat pups (Fig. 8A). Sevoflurane exposure (3%, 6 h) was carried out at PND 7. For cognitive assessment, the MWM and fear conditioning test were conducted to assess the hippocampus-dependent spatial and emotional cognitive deficits in developmental sevoflurane neurotoxicity. The open field test, MWM and fear conditioning experiments were performed at PND 36, 39 and 46, respectively. The behavior protocol is shown in Fig. 8B. In open field test, the travelled distances were indistinguishable among groups (Fig. 8C), suggesting that the locomotor activity is not responsible for the cognitive differences.

At place trial day 3 and 4 of MWM, the sevoflurane exposed animals spent significantly more time to find the platform than the animals of control group, while the animals injected with AAV-delivered cadRNAs before sevoflurane exposure were behaved similar compared with the control rats (Fig. 8D). In probe test of MWM, the sevoflurane exposed animals spent significantly less time in the target quadrant than the animals of control group, while the animals delivered with cadRNAs before sevoflurane treatment were indistinguishable from the animals of control group (Fig. 8E). Furthermore, the number of crossings was significantly increased in Sevo + cadRNAs group when compared with the sevoflurane group (Fig. 8F). In fear conditioning experiment, the freezing response to the context and cued fear conditioning of Sevo + cadRNAs group were significantly improved when compared with the animals of sevoflurane group (Fig. 8G-H). Together, these results implied that the spatial and emotional deficits induced by developmental sevoflurane exposure could be restored by AAV-delivered cadRNAs.

## Discussion

Owing to the pharmacological features of low blood-gas partition coefficient along with less irritation to the airway, sevoflurane is widely used among neonate, infants, and children (Lerman et al. 1994). In the present study, we showed that sevoflurane priming leads to neuronal death and transcriptional alternation in the developing brain. Concomitantly, sevoflurane exposure initiates PANoptosis and inflammatory response. In addition, we provided the evidence that ADAR1-P150 inhibits PANoptosis and inflammatory response through competing with ZBP1 for binding to Z-RNA in sevoflurane neurotoxicity. Furthermore, ADAR1 A-to-I RNA editing improves sevoflurane-induced cell death and PANoptosis as well as Z-RNA production. Finally, delivery of cadRNAs substantially ameliorates sevoflurane-induced neurocognitive deficits. As far as we know, this is the initial study to identify the role of neuronal PANoptosis in developmental anesthetic neurotoxicity.

In the past decades, a great deal of evidence supports the viewpoint that neuronal apoptosis mainly contributes to anesthetic neurotoxicity during synaptogenesis. However, accumulating evidence suggests that the release of inflammatory cytokines are also implicated in developmental anesthetic neurotoxicity. For example, sevoflurane priming gives rise to an increase of IFN-γ, IL-1β and TNF-α (Tang et al. 2021; Wali et al. 2022). Anesthetic-induced inflammatory profiles can not be explained by cell apoptosis alone, because apoptosis is supposed to be a non-inflammatory form of cell death. Unlike apoptosis, both pyroptosis and necroptosis are driven by dedicated membrane pore-forming proteins, and are considered as inflammatory types of cell death. In this study, we showed that GSDMD-NT, GSDME-NT, cleaved Caspase-3, cleaved Caspase-7, RIPK3 and P-MLKL are elevated by sevoflurane priming, confirming that PANoptosis was strongly implicated in developmental sevoflurane neurotoxicity. More importantly, we found that ADAR1 is capable of suppressing ZBP1-dependent cellular PANoptosis and inflammatory responses in sevoflurane neurotoxicity, in agreement with a recent study stating that ADAR1 deficiency sensitizes cells to ZBP1-induced cellular PANoptosis triggered by nuclear export inhibition (Karki et al. 2021). These evidences have expanded our understanding of the new functions of ADAR1 and ZBP1 beyond their classical roles.

Plasma membrane rupture is proposed as the final cataclysmic event in lytic cell death. Ruptured cells unleashes damage-associated molecular patterns (DAMPs) to propagate inflammatory responses. As DAMPs, intracellular smaller proteins, such as IL-18 (18 kDa) or IL-1β (17 kDa), are permitted to pass via pyroptotic pores. In contrast, larger proteins such as HMGB1 (tetramer of 150 kDa) or LDH (140 kDa) are not allowed to secrete through pyroptotic pores (Volchuk et al. 2020). Instead, these bigger inflammatory mediators are spilled after cell lysis following cellular content release. The protein NINJ1 has been considered to be a terminal executor of plasma membrane rupture during cell death, because NINJ1-deficient cells are resistant to discharge intracellular proteins including LDH and HMGB1. As a potent signaling molecule, HMGB1 is discharged into the extracellular space in response to many stimuli (e.g. developmental sevoflurane exposure), resulting in the release of pro-inflammatory chemokines and cytokines. Therefore, we proposed that sevoflurane exposure triggers ZBP1-mediated PANoptosis and subsequent plasma membrane rupture, which facilitates the release of inflammatory effectors including IL-1β, IL-18, TNF-α and IFN-γ, and NINJ1-mediated membrane rupture and subsequent HMGB1 release play a critical role in this process.

Interestingly, the neuronal protective effects of A-to-I editing on sevoflurane treatment could be almost abolished by ADAR1-P150 shRNA transfection, intimating that this effect is predominantly mediated by ADAR1-P150. Utilizing isoform-specific editome analysis, Tony et al. recently found that more than half of the A-to-I edit sites are selectively edited by ADAR1-P150, and the other half are edited by either ADAR1-P150 or ADAR1-P110 (Sun et al. 2021). Moreover, this team also reported that the ADAR1-P150 mRNA is capable of co-expressing both ADAR1-P150 and ADAR1-P110 isoforms. Although ADAR2 has more targeted expression to tissues including brain, and is responsible for the high editing rates in neuronal tissues, ADAR2 has no Zα domain to interact with ZBP1 or Z-NAs. ADAR3 is brain specific and has yet to demonstrate detectable RNA editing activity. In our study, sevoflurane treatment promoted the expression of ADAR3, and may consequently suppress the ADAR1 activity in the brain (Raghava Kurup et al. 2022). These findings are support of the viewpoint that ADAR1-P150 is essential for ADAR1 dependent A-to-I RNA editing.

In the model of sevoflurane neurotoxicity, we have shown that the overall diminished A-to-I RNA editing is accompanied by cellular accumulation of Z-RNAs, which was detected by Z22 antibody and confirmed by RNase A and RNase H. One possible interpretation for Z-RNAs accumulation is that ADAR1 loss-of-function results in impaired editing of endogenous retroelement (ERE)-derived complementary RNA reads, leading to the upregulation of dsRNAs with the capacity to generate Z-RNAs (Jiao et al. 2022). On the other hand, ADAR1-dependent editing of Alu repeats may compromise the stability of the Z-RNA duplexes that they form (de Reuver et al. 2022). Since the majority of elements edited by ADAR1 are Alu repeats, it is conceivable that such Alu-Alu duplexes would be more stable and susceptible to activate ZBP1 in the scenario of sevoflurane-induced ADAR1 deficiency.

Delivery of cadRNAs significantly attenuates PANoptosis, inflammatory response and subsequent cognitive deficits induced by sevoflurane exposure. Of note, the approach of engineered cadRNAs delivery, which recruits endogenous ADAR1 (Katrekar et al. 2022), is distinct from enzyme overexpression. One limitation of enzyme overexpression is the introduction of excessive off-target A-to-I edits across the transcriptome. Since ADARs are native to most mammalian systems, their overexpression may lead to other protein interactions that might exert negative effects on normal cellular process. Furthermore, a recent study showed that AAV-delivered cadRNAs yielded much higher levels of targeted editing than their linear ADAR-recruiting guide RNAs, and in a long-lasting (up to 21 days) fashion (Yi et al. 2022). As cadRNAs do not need for co-delivery of any other effector proteins, and RNA editing is reversible and tunable without causing permanent changes in the genome, the molecular mechanisms identified here are suitable for the improvement of sevoflurane neurotoxicity, especially for in vivo applications.

**Fig. 9:**
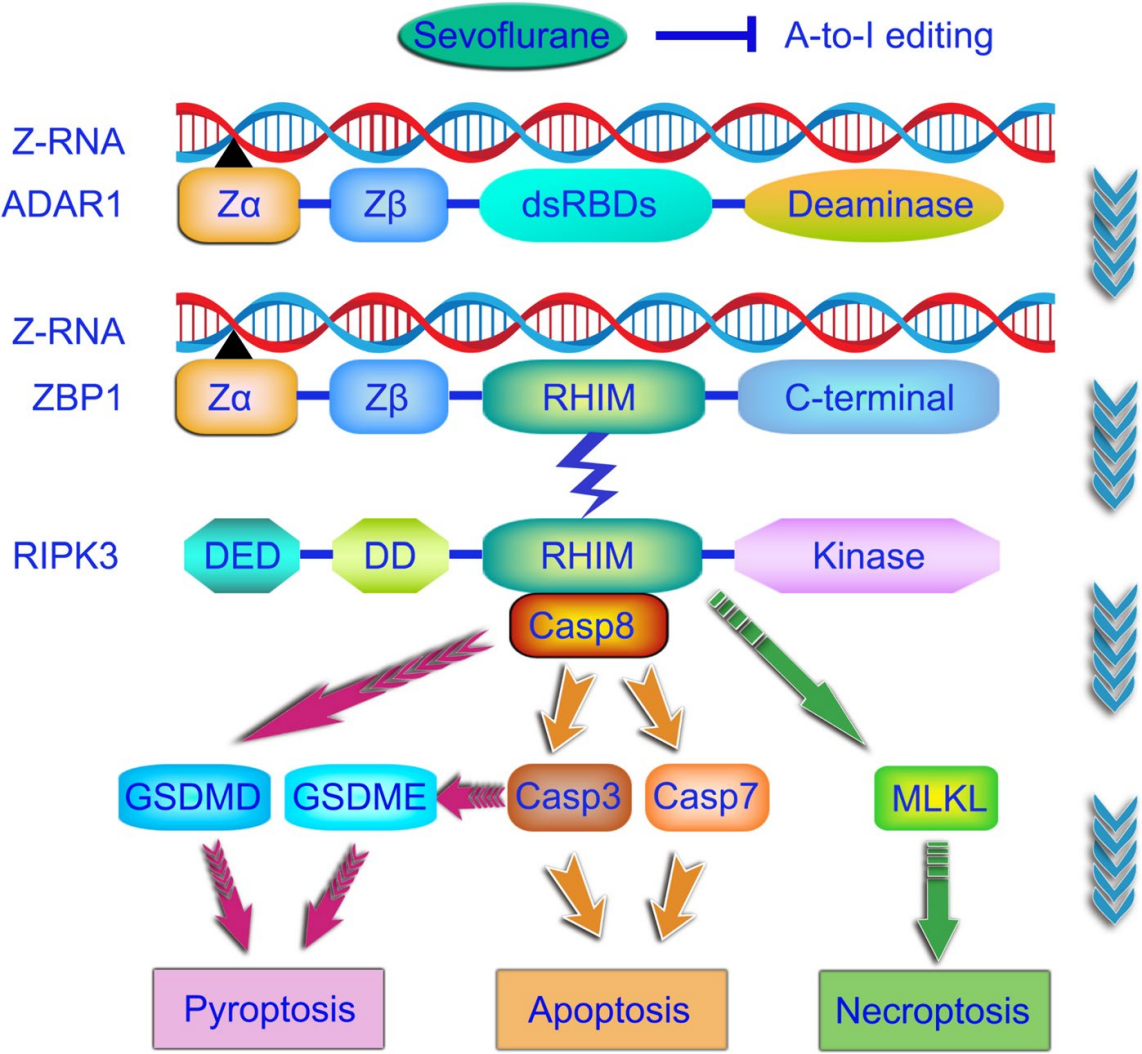
Inhibition of ZBP1-dependent PANoptosis via ADAR1 A-to-I RNA editing in sevoflurane neurotoxicity. In physiological condition, structural modification of RNA by ADAR1 A-to-I editing through binding to its Zα domain renders self-RNA invisible to RNA sensors. Lack of A-to-I RNA editing induced by sevoflurane exposure results in cellular Z-RNAs accumulation and aberrant ZBP1 activation. RIPK3 is subsequently activated in a RHIM-dependent manner. RIPK3 interacting with Caspase-8 leads to GSDMD-dependent cell pyroptosis, and activation of Caspase-3 and Caspase-7. Additionally, GSDME can switch Caspase-3-mediated apoptosis to pyroptosis. Moreover, activated RIPK3 can trigger MLKL-mediated neuronal necroptosis in the scenario of sevoflurane. Therefore, the orchestration of PANoptosis is carefully tuned to control cell fate in response to developmental sevoflurane stimuli. Abbreviations: A-to-I, adenosine-to-inosine; DD, death domain; DED, death effector domain; dsRBDs, double-stranded RNA-binding domains; GSDMD, gasdermin D; GSDME, gasdermin E; MLKL, mixed lineage kinase-like; RHIM, RIP homotypic interaction motif

Physiologically, structural modification of RNA by ADAR1 A-to-I editing through binding to its Zα domain renders self-RNA invisible to RNA sensors. Sevoflurane-induced lack of RNA editing results in cellular Z-RNAs accumulation and ZBP1 aberrant activation. Coupled with Caspase-8, RIPK3 is activated in a RIP homotypic interaction motif (RHIM)-dependent manner, triggering GSDMD-dependent cell pyroptosis and Caspases cascade. Additionally, GSDME is capable of switching Caspase-3-mediated apoptosis to pyroptosis (Wang et al. 2017). RIPK3-dependent and MLKL-mediated neuronal necroptosis was also identified in developmental sevoflurane neurotoxicity (Wang et al. 2022; Xu et al. 2022). Therefore, the orchestration of PANoptosis is carefully tuned to regulate cell fate in response to sevoflurane stimuli as shown in Fig. 9. Our findings also provide mechanistic insights into how ADAR1 and ZBP1, and their interactions, are implicated in inflammatory initiation and subsequent cognitive dysfunctions. On one hand, sevoflurane-induced production of pro-inflammatory cytokines (e.g. TNF-α, IL-1β, IL-18 and IFN-γ) are capable of directly unleashing inflammatory response. On the other hand, ZBP1-mediated PANoptosis may indirectly contribute to detrimental inflammatory responses via the release of DAMPs (e.g. HMGB1 and TNF-α) from dying cells.

To fully appreciate these findings, there are several points should be considered. Firstly, the neuroprotective role of cadRNAs delivery is mainly contributed to the restoration of ADAR1-P150 RNA editing activity and subsequent relief of ZBP1-mediated PANoptosis induced by developmental sevoflurane exposure. For RNA editing of adenosines, however, the activity of other members of ADARs family, such as ADAR1-P110 and ADAR2, can also be enhanced by engineered cadRNAs application. These ADAR members may also partially contributed to A-to-I RNA editing and Z-RNA accumulation. As to cognitive functions, the relief of neuronal loss accounts for the amelioration of spatial and emotional impairments. Nevertheless, the attenuation of DAMPs release may also implicate in the improvements of cognitive disorders. For example, HMGB1 is reported to mediate the memory decline in septic model (Chavan et al. 2012), and is correlated with cognitive dysfunction in the survivors of intensive care unit (Bruck and Lasselin 2020). Besides this, aberrant activation of TNF-α can cause persistent synaptic alteration and the formation of fear memory (Habbas et al. 2015; Yu et al. 2017).

In summary, we have demonstrated that the ZBP1-mediated PANoptosis and inflammation are critically involved in sevoflurane neurotoxicity. We also identified the role of ADAR1-P150 dependent A-to-I RNA editing in this process. Additionally, AAV-cadRNAs delivery substantially improved the spatial and emotional cognitive deficits. Consequently, delivery of engineered cadRNAs to rectify the compromised ADAR1-dependent A-to-I RNA editing is an inspiring direction to prevent developmental anesthetic neurotoxicity.

## Supplementary Information

Below is the link to the electronic supplementary material.

Supplementary file1 Supplemental Fig. 1 Transfection efficiency of ADAR1 P150 plasmid and ZBP1 shRNA in vivo. A Entranster‐in vivo transfection reagent (10 μl) was added to 5 μg ADAR1-P150 plasmid or 5 μg empty vectors. The solution was then mixed at 25℃ for 15 min. Entranster‐in vivo-plasmid mixtures were injected intracerebroventricularly. After 48 h, the hippocampus was collected for determine the expression of ADAR1 P150. B To knock down ZBP1 expression in vivo, RNA interference was applied using the shRNA against ZBP1. The ZBP1 shRNA (500 pmol) or scrambled shRNA (500 pmol) were dissolved in 5 μl RNase‐free water. The Entranster‐in vivo RNA transfection reagents (10 μl) were added to 5 μl shRNA or 5 μl scrambled shRNA. After mixing for 15 min at 25℃, Entranster-in vivo-siRNA mixture was injected intracerebroventricularly. After 48 h, the hippocampus was collected for determine the expression of ZBP1. (JPG 402 KB)

Supplementary file2 Supplemental Fig. 2 The neurocognitive functions of rats injected with scrambled RNA. A The locomotor activity was examined as the total distance traveled in 10 min to measure the possible locomotor activity impairments. B In place trials of MWM, latency was defined as the time to reach the submerged platform. C In probe test of MWM, the time spend in targeted quadrant was analyzed. D In probe test of MWM, the number of crossings were examined. E The histogram shows the percentage of freezing time 24 h after conditioning. F The histogram represents the percentage of freezing time in cued test 48 h after conditioning. (JPG 896 KB)

## Data Availability

No datasets were generated or analysed during the current study.

## Abbreviations

ADAR1: Adenosine deaminase acting on the RNA-1
A-to-I: Adenosine-to-Inosine
cadRNAs: Circular ADAR-recruiting guide RNAs
DAMPs: Damage-associated molecular patterns
dsRNAs: Double-stranded RNAs
HMGB1: High-mobility group box 1
IFN-γ: Interferon γ
IL-18: Interleukin-18
IL-1β: Interleukin-1β
LDH: Lactate dehydrogenase
MWM: Morris water maze
MLKL: Mixed lineage kinase domain-like protein
NINJ1: Ninjurin-1
PANoptosis: Pyroptosis, apoptosis and necroptosis
RIPK3: Receptor-interacting protein kinase-3
TNF-α: Tumor necrosis factor-α
ZBP1: Z-DNA/RNA binding protein 1
Z-NAs: Z-nucleic acids
