# Supplementary figures and images for: ADAR1 prevents ZBP1-dependent PANoptosis via A-to-I RNA editing in developmental sevoflurane neurotoxicity
Source: Cell Biol Toxicol. 2024 Jul 25;40(1):57. doi: 10.1007/s10565-024-09905-1 (PMC11281990; doi:10.1007/s10565-024-09905-1)

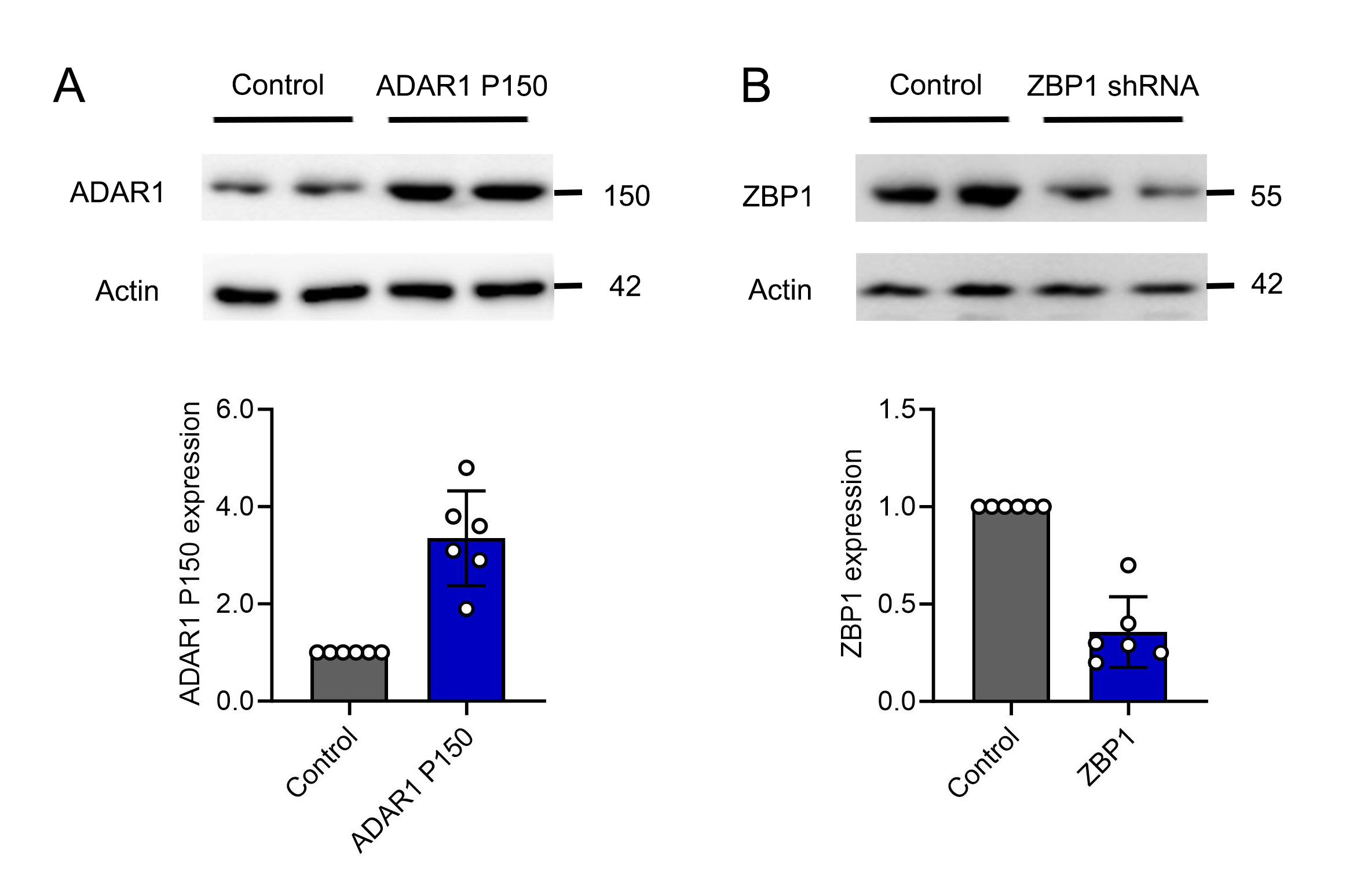

Supplement: Supplementary file 1 — Supplementary file1 Supplemental Fig. 1 Transfection efficiency of ADAR1 P150 plasmid and ZBP1 shRNA in vivo. A Entranster‐in vivo transfection reagent (10 μl) was added to 5 μg ADAR1-P150 plasmid or 5 μg empty vectors. The solution was then mixed at 25℃ for 15 min. Entranster‐in vivo-plasmid mixtures were injected intracerebroventricularly. After 48 h, the hippocampus was collected for determine the expression of ADAR1 P150. B To knock down ZBP1 expression in vivo, RNA interference was applied using the shRNA against ZBP1. The ZBP1 shRNA (500 pmol) or scrambled shRNA (500 pmol) were dissolved in 5 μl RNase‐free water. The Entranster‐in vivo RNA transfection reagents (10 μl) were added to 5 μl shRNA or 5 μl scrambled shRNA. After mixing for 15 min at 25℃, Entranster-in vivo-siRNA mixture was injected intracerebroventricularly. After 48 h, the hippocampus was collected for determine the expression of ZBP1. (JPG 402 KB) [file 10565_2024_9905_MOESM1_ESM.jpg]

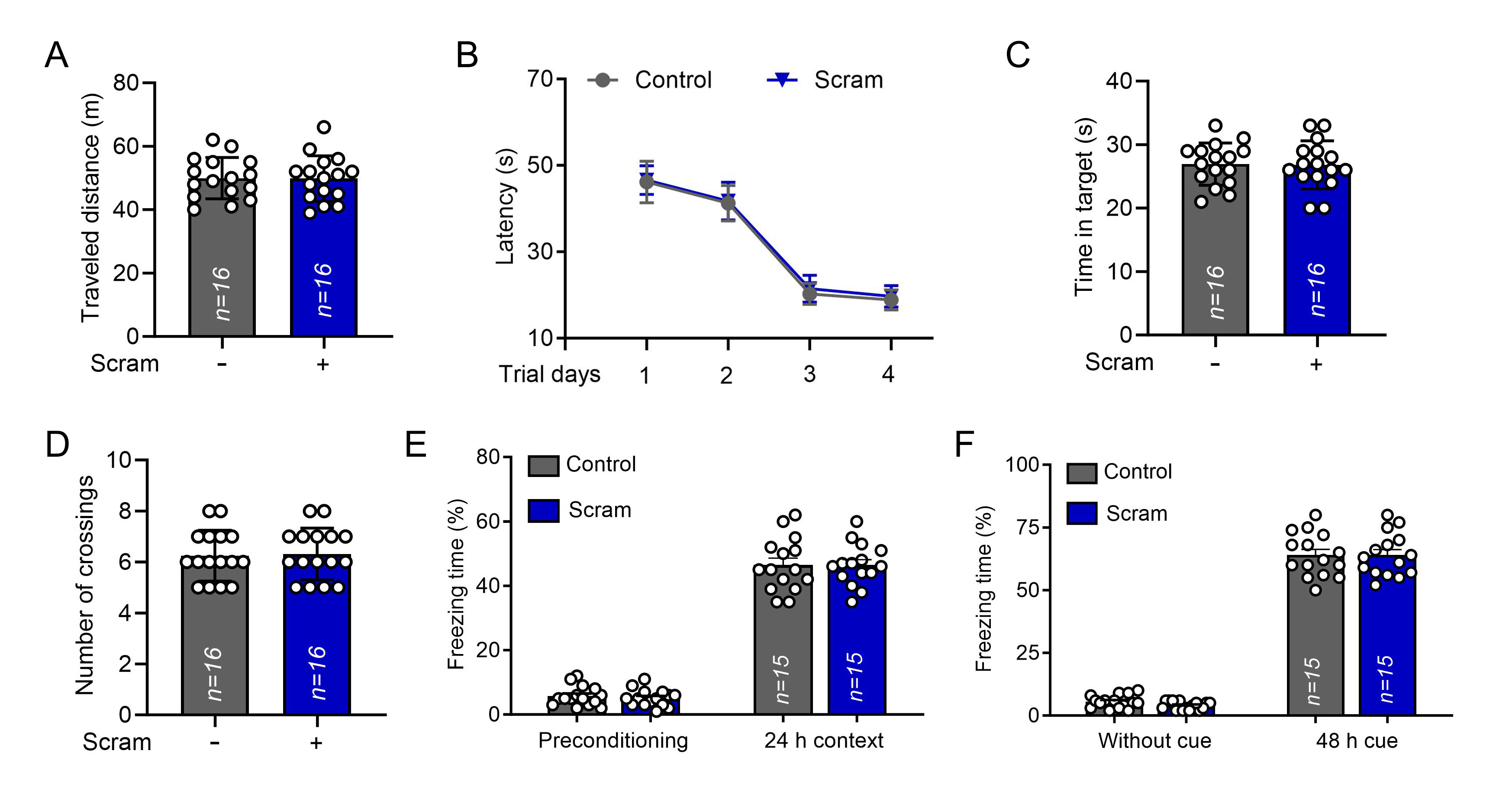

Supplement: Supplementary file 2 — Supplementary file2 Supplemental Fig. 2 The neurocognitive functions of rats injected with scrambled RNA. A The locomotor activity was examined as the total distance traveled in 10 min to measure the possible locomotor activity impairments. B In place trials of MWM, latency was defined as the time to reach the submerged platform. C In probe test of MWM, the time spend in targeted quadrant was analyzed. D In probe test of MWM, the number of crossings were examined. E The histogram shows the percentage of freezing time 24 h after conditioning. F The histogram represents the percentage of freezing time in cued test 48 h after conditioning. (JPG 896 KB) [file 10565_2024_9905_MOESM2_ESM.jpg]
